# Phenylethanol glycosides from the seeds of *Aesculus chinensis* var. *chekiangensis*

**DOI:** 10.1186/s13065-020-00685-3

**Published:** 2020-04-22

**Authors:** Nan Zhang, Di Liu, Shuxiang Wei, Shijie Cao, Xinchi Feng, Kai Wang, Liqin Ding, Feng Qiu

**Affiliations:** 1 School of Chinese Materia Medica, Tianjin University of Traditional Chinese Medicine, No. 10 Poyanghu Road, West Area, Tuanbo New Town, Jinghai Dist, Tianjin, 301617 People’s Republic of China; 2 Tianjin State Key Laboratory of Modern Chinese Medicine, Tianjin University of Traditional Chinese Medicine, Tianjin, China

**Keywords:** *Aesculus chinensis* Bge. var. *chekiangensis* (Hu et Fang) Fang, Phenylethanol glycosides, Neuroprotective activity

## Abstract

Three new phenylethanol glycosides (**1**-**3**) and one known analogue (**4**) were isolated from the seeds of *Aesculus chinensis* Bge. var. *chekiangensis*. To the best of our knowledge, this represents the first isolation of phenylethanol glycosides from the genus of *Aesculus*, which enriched its chemical composition. Structure elucidations were performed via extensive NMR and HRESIMS data together with comparison with literature data. Thereafter, the isolated compounds were assayed for their neuroprotective activities against CoCl_2_-induced cytotoxicity in PC12 cells and compound **3** exhibited moderate activity.

## Introduction

**Fig. 1:**
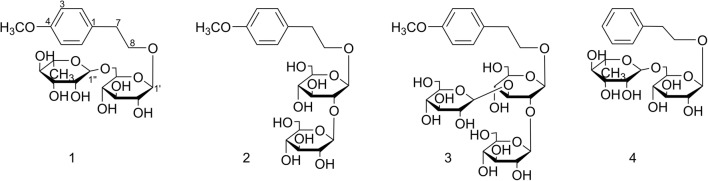
The structures of isolated compounds

The genus *Aesculus*, which belongs to the family *Hippocastanaceae* contains about 30 species found worldwide. The dried seeds of *Aesculus chinensis* Bge. var. *chekiangensis* (Hu et Fang) Fang, *Aesculus chinensis* Bge and *Aesculus wilsonii* Rehd are commonly used to treat chest and abdomen pain, dysentery and ague [1, 2] in traditional Chinese medicine. Previous studies on the genus of *Aesculus* revealed the presences of diverse secondary metabolites such as triterpenoids [3–7], flavonoids [8, 9], coumarins [10] and steroids [11]. And a number of pharmacological studies have suggested that *A. chinensis* exhibited beneficial effects on antitumor [12], neuroprotective [13], anti-inflammatory [14] and cardio-protective activities [15]. Nevertheless, compared to other species of *Aesculus* genus, the chemical investigation of *Aesculus chinensis* Bge. var. *chekiangensis* (Hu et Fang) Fang is limited. Our interests in cytotoxic and neuroprotective components from *A. chinensis* Bge. var. *chekiangensis* (Hu et Fang) Fang have led to the isolation of numerous new ones [16, 17]. As a continuous search for structurally novel compounds with diverse bioactivities, three new phenylethanol glycosides (**1**-**3**) and one known analog (**4**) were obtained (Fig. 1), which represent the first examples of phenylethanol glycosides obtained from the genus of *Aesculus*. Herein, the isolation, structure identification and biological evaluation of **1-4** are described.

## Methods

### General experimental procedures

The chemicals and material were similar to our previous researches [16, 17].

### Plant material

The plant was the same batch of medicinal material as our previous reports [16, 17].

### Extraction and isolation

The extracted method was the same to our previous studies [16, 17]. Chopped, dried seeds of *A. chinensis* Bge. (8.8 kg) were extracted with 70% ethanol, then partitioned via D101 resin column eluting with a stepwise gradient of H_2_O-EtOH.

The 60% EtOH-H_2_O part was loaded onto a silica gel column using CH_2_Cl_2_/CH_3_OH (100:1 → 1:1) to yield 4 fractions (A–D). Fraction A was further separated by RP C_18_ CC (MeOH–H_2_O, from 0:100 to 100:0) to give four subfractions (A1–A4). Subfraction A2 was chromatographed over a Sephadex LH-20 column (MeOH) then RP-HPLC (MeOH–H_2_O, 35:65, 3.0 mL/min) to give compounds **1** (11.0 mg) and **4** (20.0 mg). Subfraction A3 was further subdivided with an ODS RP-C18 column (MeOH/H_2_O, 10:90 to 100: 0) to give seven subfractions (A3A–A3G). The subfraction A3G was applied to a Sephadex LH-20 column (MeOH), and then purified by recycling preparative HPLC with 40% MeOH/H_2_O to yield compounds **2** (3.7 mg) and **3** (9.0 mg).

#### 4-methoxy-phenylethanol-8-*O*-*α*-l-rhamnopyranosyl-(1 → 6)-*β*-d-glucopyranoside (1)

Brown amorphous powder; [*α*]25 *D*–7.3 (*c* 0.10, MeOH); Proton nuclear magnetic resonance (^1^H-NMR) and carbon-13 nuclear magnetic resonance (^13^C-NMR): Table 1; HR-ESI–MS: *m/z* 505.1918 [M + COOH]^−^ (calculated for C_22_H_33_O_13_, 505.1921).

**Table 1:** ^1^H (600 MHz) and ^13^C (150 MHz) NMR data of **1**–**3** (*δ* in ppm, in DMSO-*d*_6_)

Position	1		2		3	
	*δ*_C_	*δ*_H_	*δ*_C_	*δ*_H_	*δ*_C_	*δ*_H_
1	130.5		130.7		130.6	
2	129.9	7.17 (1H, d, *J *= 8.5 Hz)	130.0	7.17 (1H, d, *J* = 8.7 Hz)	130.0	7.18 (1H, d, *J *= 8.7 Hz)
3	113.7	6.83 (1H, d, *J *= 8.5 Hz)	113.6	6.83 (1H, d, *J* = 8.7 Hz)	113.6	6.82 (1H, d, *J* = 8.7 Hz)
4	157.6		157.6		157.7	
5	113.7	6.83 (1H, d, *J *= 8.5 Hz)	113.6	6.83 (1H, d, *J* = 8.7 Hz)	113.6	6.82 (1H, d, *J* = 8.7 Hz)
6	129.9	7.17 (1H, d, *J *= 8.5 Hz)	130.0	7.17 (1H, d, *J* = 8.7 Hz)	130.0	7.18 (1H, d, *J *= 8.7 Hz)
7	34.8	2.78 (2H, dt, *J *= 8.2, 6.3 Hz)	34.7	2.78 (2H, dt, *J *= 8.2, 6.4 Hz)	34.6	2.78 (2H, dt, *J *= 8.4, 6.6 Hz)
8	69.9	3.83 (1H, dt, *J *= 8.2, 6.3 Hz) 3.62 (1H, overlap)	69.8	3.89 (1H, dt, *J *= 8.2, 6.4 Hz) 3.60 (1H, overlap)	69.9	3.92 (1H, dt, *J *= 8.4, 6.6 Hz) 3.60 (1H, overlap)
4-OCH_3_	55.0	3.71 (3H, s)	55.0	3.71 (3H, s)	54.9	3.71 (3H, s)
1′	103.0	4.17 (1H, d, *J *= 7.7 Hz)	101.3	4.33 (1H, d, *J *= 7.7 Hz)	101.6	4.39 (1H, d, *J *= 7.5 Hz)
2′	73.4	2.94 (1H, t, *J *= 8.5 Hz)	82.2	3.23 (1H, dd, *J *= 9.1, 7.7 Hz)	79.6	3.44 (1H, m)
3′	76.7	3.13 (1H, t, *J *= 8.9 Hz)	76.7	3.10 (1H, m)	86.2	3.49 (1H, t, *J *= 8.8 Hz)
4′	70.2	2.99 (1H, t, *J *= 9.1 Hz)	69.8	3.10 (1H, m)	68.4	3.19 (1H, m)
5′	75.4	3.26 (1H, m)	76.1	3.36 (1H, m)	76.1	3.18 (1H, m)
6′	67.0	3.81 (1H, dd, *J *= 11.2, 2.0 Hz) 3.42 (1H, dd, *J *= 11.2, 6.0 Hz)	60.9	3.65 (1H, m) 3.42 (1H, dd, *J *= 11.2, 5.2 Hz)	61.2	3.65 (1H, m) 3.47 (1H, m)
1′′	100.8	4.59 (1H, d, *J *= 1.2 Hz)	104.1	4.41 (1H, d, *J *= 7.8 Hz)	102.8	4.55 (1H, d, *J *= 8.0 Hz)
2′’	70.5	3.60 (1H, m)	74.9	3.00 (1H, dd, *J *= 8.4, 7.8 Hz)	74.5	2.94 (1H, dd, *J *= 8.5, 5.9 Hz)
3′′	70.7	3.42 (1H, m)	77.1	3.08 (1H, m)	76.4	3.19 (1H, m)
4′′	72.0	3.17 (1H, t, *J *= 9.1 Hz)	69.9	3.10 (1H, m)	70.0	3.62 (1H, m)
5′’	68.4	3.45 (1H, m)	76.1	3.16 (1H, m)	76.2	3.16 (1H, m)
6′′	18.0	1.12 (3H, d, *J *= 6.2 Hz)	61.0	3.65 (1H, m) 3.49 (1H, dd, *J *= 11.2, 5.5 Hz)	61.0	3.67 (1H, m) 3.39 (1H, dd, *J *= 11.8, 5.9 Hz)
1′′′					103.4	4.38 (1H, d, *J *= 7.9 Hz)
2′′′					73.7	3.04 (1H, d, *J *= 8.7 Hz)
3′′′					76.9	3.07 (1H, m)
4′′′					70.1	3.06 (1H, m)
5′′′					76.8	3.05 (1H, m)
6′′′					60.7	3.69 (1H, m) 3.45 (1H, m)

#### 4-methoxy-phenylethanol-8-*O*-*β*-d-glucopyranosyl-(1 → 2)-*β*-d-glucopyranoside (2)

Brown amorphous powder; [*α*]25 *D*–11.2 (*c* 0.11, MeOH); ^1^H-NMR and ^13^C-NMR: Table 1; HR-ESI–MS: *m/z* 521.1870 [M + COOH]^−^ (calculated for C_22_H_33_O_14_, 521.1870).

#### 4-methoxy-phenylethanol-8-*O*-*β*-d-glucopyranosyl-(1 → 2)-[*β*-d-glucopyranosyl-(1 → 3)]-*β*-d-glucopyranoside (3)

Brown amorphous powder; [*α*]25 *D*–14.6 (*c* 0.10, MeOH); ^1^H-NMR and ^13^C-NMR: Table 1; HR-ESI–MS: *m/z* 683.2398 [M + COOH]^−^ (calculated for C_28_H_43_O_19_, 683.2399).

### Hydrolysis and determination of absolute configuration of sugars

Compounds **1**–**3** (1.0 mg, respectively) was hydrolyzed with 2 M HCl (4.0 mL) at 90 °C for 2 h. Then the hydrolysed materials were disposed and tested by means of the procedure described in our previous work [16, 17].

### Neuroprotective effect assay

Compounds **1**-**4** were assayed for their neuroprotective effects against CoCl_2_-induced PC12 cell injury [18] by 3-(4,5-dimethylthiazol)-2,5-diphenyltetrazolium bromide (MTT) method with trolox as the positive control according to our previously reported procedure [16, 17]. PC12 cells were cultured in RPMI-1640 medium containing 10% fetal bovine serum as well as 100 U/mL penicillin/streptomycin and were incubated at 37 °C with 5% CO_2_. PC12 cells were placed into a 96-well plate at a density of 2 × 10^4^ cells/well and kept there for 24 h. Cells were incubated with test compounds and trolox (10 μM) for 2 h. To induce an oxidative stress, 1 mM CoCl_2_ was added to the cells and incubated for 24 h. Then, the supernatant was changed with 100 μL MTT solution (5 mg/mL) for 2.5 h, the plate was vibrated, and the absorbance at 490 nm was measured using a microplate reader.

### Cytotoxicity assay

Cell viability was determined with the MTT method [19, 20]. The human hepatocellular carcinomas cells (HepG2), the human colorectal carcinoma cells (HCT-116) and the human gastric carcinoma cells (MGC-803) were purchased from ATCC. HepG2, MGC-803 and HCT-116 were respectively cultured in DMEM and RPMI-1640 mediums, which were supplemented with 10% fetal bovine serum at 37 °C in a humidified atmosphere containing 5% CO_2_. HepG2, HCT-116, and MGC-803 cells (1 × 10^4^) were seeded in 96-well tissue culture plates. Cells were treated in triplicate with five concentrations (50, 25, 12.5, 6.25 and 3.125 μM) of the tested compounds for 24 h, with 5-fluorouracil (5-FU) as positive control. Subsequently, 100 μL of MTT (5 mg/mL) was added and the cells were incubated for additional 2.5 h. Thereafter, the supernatant was discarded and 0.15 ml of DMSO was added to each well, then the plate was mixed on a microshaker for 10 min and read on a microplate reader at 490 nm.

## Results and discussion

**Fig. 2:**
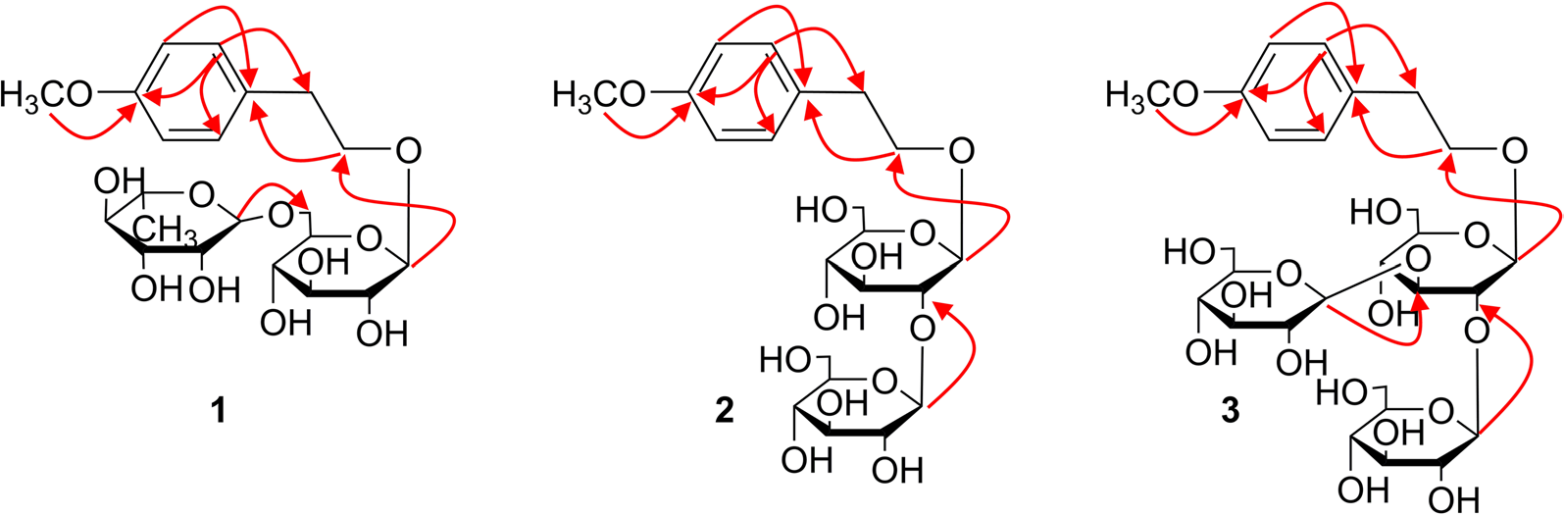
Selected HMBC (H → C) correlations of compounds **1-3**

Compound **1** was obtained as brown amorphous powder with a molecular formula of C_21_H_32_O_11_ deduced from its HR-ESI-MS spectrum (*m/z* 505.1918 [M + COOH]^−^, calcd. for C_22_H_33_O_13_, 505.1921). The ^1^H-NMR spectrum of compound **1** exhibited signals characteristic for a 1, 4-disubstituted benzene ring [*δ*_H_ 7.17 (2H, d, *J* = 8.5 Hz, H-2, 6), 6.83 (2H, d, *J* = 8.5 Hz, H-3, 5)], an ethoxy moiety [*δ*_H_ 2.78 (2H, dt, *J *= 8.2, 6.3 Hz), 3.83 (1H, dt, *J *= 8.2, 6.3 Hz)] as well as a *O*-methyl at *δ*_H_ 3.71 (3H, s) (Table 1). The heteronuclear multiple bond correlations (HMBC) (Fig. 2) of H-2 (*δ*_H_ 7.17) to C-4 (*δ*_C_ 157.6), C-6 (*δ*_C_ 129.9), C-7 (*δ*_C_ 34.8); H-3 (*δ*_H_ 6.83) to C-1 (*δ*_C_ 130.5), C-5 (*δ*_C_ 113.7); H-8 (*δ*_H_ 3.83) to C-1 (*δ*_C_ 130.5) and OCH_3_ (*δ*_H_ 3.71) to C-4 (*δ*_C_ 157.6) indicated **1** contains a 4-methoxy-phenylethanol moiety.

The two anomeric protons at *δ* 4.17 (1H, d, *J *= 7.7 Hz), 4.59 (1H, d, *J *= 1.2 Hz) correlated with carbons at *δ* 103.0 and 100.8 in heteronuclear single quantum coherence (HSQC) spectrum, respectively, indicated a disaccharide residue. Acid hydrolysis of **1** liberated d-glucose and l-rhamnose, which were identified by HPLC analysis after derivatization [21, 22]. The *β*-orientation of the glucopyranosyl unit was deduced from the coupling constant (*J* = 7.7 Hz, H-1′). The *α*- anomeric configuration of rhamnose was determined from the absence of nuclear overhauser effect spectroscopy (NOESY) correlations between protons H‑1 and H‑3/H‑5. The *β*-d-glucose was attached to the 4-methoxy-phenylethanol nucleus at C-8, evidenced by the HMBC correlation between H-1′ (*δ*_H_ 4.17) to C-8 (*δ*_C_ 69.9). In addition, the downfield chemical shift of C-6′ (*δ*_C_ 67.0) of the glucose coupled with the cross peak of H-1′′ (*δ*_H_ 4.59) to C-6′ (*δ*_C_ 67.0) in HMBC spectrum suggesting the *α*-l-rhamnose was linked to C-6′. Based on these data, compound **1** was concluded to be 4-methoxy-phenylethanol‐8-*O*-*α*-l-rhamnopyranosyl-(1 → 6)-*β*-d-glucopyranoside.

The elemental formula of compound **2** was confirmed to be C_21_H_32_O_12_ with one oxygen more than that of **1** according to the [M + COOH]^−^ ion peak at *m/z* 521.1870 in its HRESIMS spectrum. The ^1^H and ^13^C NMR data of **2** revealed a close resemblance to **1** except for the corresponding signals to the two sugar units. Careful analysis of the NMR data and the acid hydrolysis results affirmed the existence of two *β*-d-glucose groups in **2** instead of one *β*-d-glucose and one *α*-l-rhamnose in **1**. HMBC correlations from H-1′ (*δ*_H_ 4.33) to C-8 (*δ*_C_ 69.8) and H-1′′ (*δ*_H_ 4.41) to C-2′ (*δ*_C_ 82.8) revealed the position and sequences of the sugar moiety in **2** as shown in Fig. 2. Hence, compound **2** was assigned as 4-methoxy-phenylethanol-8-*O*-*β*-d-glucopyranosyl-(1 → 2)-*β*-d-glucopyranoside.

Compound **3** was also acquired as a brown solid with the molecular formula of C_27_H_42_O_17_ (*m/z* 683.2398 [M + COOH]^−^; calcd. for C_28_H_43_O_19_, 683.2399), which is 162 mass units more than that of **2**. The NMR data of **3** were closely resemble to those of **2**, indicating the same aglycone with the difference of an additional hexose moiety. d-glucose was afforded from **3** via the same procedure as before and the *β* configuration was inferred from the large coupling constants: [*δ*_H_ 4.39 (1H, d, *J* = 7.5 Hz, H-1′), 4.55 (1H, d, *J* = 8.0 Hz, H-1′′), 4.38 (1H, d, *J* = 7.9 Hz, H-1′′′)]. HMBC correlations from H-1′ (*δ*_H_ 4.39) to C-8 (*δ*_C_ 69.9) supported the attachment of the sugar units to C-8. The sequence of the sugar chain was further established by the long correlations of H-1′′ (*δ*_H_ 4.55) and C-2′ (*δ*_C_ 79.6), H-1′′′ (*δ*_H_ 4.38) and C-3′ (*δ*_C_ 86.2) (Fig. 2). Consequently, compound **3** was assigned as 4-methoxy-phenylethanol-8-*O*-*β*-d-glucopyranosyl-(1 → 2)-[*β*-d-glucopyranosyl-(1 → 3)]-*β*-d-glucopyranoside.

The other known one, phenylethanol-8-*O*-*α*-l-rhamnopyranosyl-(1 → 6)-*β*-d-glucopyranoside (**4**) were also obtained and identified by NMR analysis and comparison with literature data [23].

All compounds (**1**–**4**) were tested in three human cancer cell lines, HepG2, HCT-116 and MGC-803, using 5-FU as the positive control. However, they did not show obvious cytotoxicity (IC_50_ > 50 μM).

**Fig. 3:**
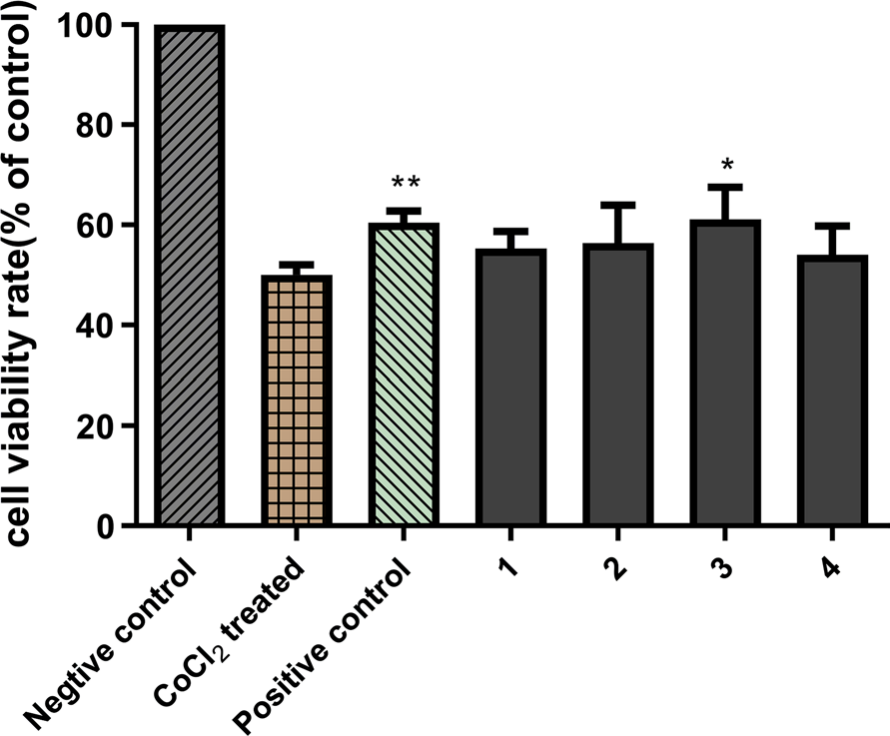
Neuroprotective activities of compounds **1-4** (10 μM) against CoCl_2_-induced cell death in PC12 cells. The data (cell viability, measured by MTT assay) are expressed as mean ± SD. Three independent experiments were performed. Trolox was used as the positive control at 10 μM. Compared with CoCl_2_ treated group, *P < 0.05, **P < 0.01

The neuroprotective effects of **1**–**4** were also evaluated in CoCl_2_-induced PC12 cell damage [24] by MTT assay. According to the references [25, 26] and our study, the positive control, trolox, exhibited statistically significant neuroprotective effect at 10 μM (Fig. 3). Therefore, the concentration of 10 μM was selected for the cytotoxic and neuroprotective evaluation of these compounds. First, the cytotoxic activity of compounds **1**–**4** against PC12 cell line was tested and none of them showed cytotoxicity at 10 μM (Additional file 1: Fig. S16). Subsequently, 10 µM compounds were bioassayed for their neuroprotective properties. And according to Fig. 3, compound **3** exhibited moderate activities against CoCl_2_-induced PC12 cell injury.

## Conclusion

In this paper, three new phenylethanol glycosides (**1-3**) and one known compound (**4**) were obtained from the seeds of *A. chinensis* Bge. var. *chekiangensis*., which represents the first isolation of phenylethanol glycosides from the genus of *Aesculus*. The findings also provided more insights into the chemotaxonomy of the *Aesculus* genus. Besides, the neuroprotective activities of the phenylethanol glycosides were also evaluated and compound **3** exhibited statistically significant neuroprotective activity.

## Supplementary information

**Additional file 1:** HR-ESI-MS, 1D- and 2D-NMR spectra of compounds **1**–**3** (**Figures S1**–**S15**), cytotoxic activities of compounds **1**–**4** on PC12 cells at 10 µM (**Figure S16**).

## Data Availability

All other datasets generated for this study are included in the article and Additional file 1.

## Abbreviations

DMSO-*d*_6_: Deuterated dimethyl sulfoxide
^1^H-NMR: Proton nuclear magnetic resonance
^13^C-NMR: Carbon-13 nuclear magnetic resonance
HMBC: Heteronuclear multiple bond correlation
HSQC: Heteronuclear single quantum coherence
NOESY: Nuclear overhauser effect spectroscopy
HepG2: The human hepatocellular carcinomas cells
HCT-116: The human colorectal carcinoma cells
MGC-803: The human gastric carcinoma cells
MTT: 3-(4,5-dimethylthiazol)-2,5-diphenyltetrazolium bromide
5-FU: 5-Fluorouracil
