# Supplementary material for: Phenylethanol glycosides from the seeds of Aesculus chinensis var. chekiangensis
Source: BMC Chem. 2020 Apr 22;14(1):31. doi: 10.1186/s13065-020-00685-3 (PMC7178748; doi:10.1186/s13065-020-00685-3)
Supplement: Supplementary file 1 — Additional file 1: HR-ESI-MS, 1D- and 2D-NMR spectra of compounds 1–3 (Figures S1–S15), cytotoxic activities of compounds 1–4 on PC12 cells at 10 µM (Figure S16). [file 13065_2020_685_MOESM1_ESM.docx]

**Phenylethanol glycosides from the seeds of *Aesculus chinensis* var.** ***chekiangensis***

Nan Zhang^#a,b^, Di Liu^#a,b^, Shuxiang Wei^a,b^, Shijie Cao^a,b^, Xinchi Feng^a^, Kai Wang^a^, Liqin Ding^b,^*, Feng Qiu^a,b,^*

*^a^* *School of Chinese Materia Medica, Tianjin University of Traditional Chinese Medicine, Tianjin, China*

*^b^ Tianjin Key Laboratory of TCM Chemistry and Analysis, Institute of Traditional Chinese Medicine, Tianjin University of Traditional Chinese Medicine, Tianjin, China*

* Corresponding authors. School of Chinese Materia Medica, Tianjin University of Traditional Chinese Medicine, No. 10 Poyanghu Road, West Area,Tuanbo New Town,Jinghai Dist, Tianjin 301617, People’s Republic of China. Phone: +86-22-59596223 E-mail: fengqiu20070118@163.com (F. Qiu), ruby70303@163.com (L.D.).

^#^ These authors contributed equally to this work.

**Supporting Information List of Contents**

| No. | Content | Page |
| --- | --- | --- |
| 1 | **Figure S1.** The HR-ESI-MS Spectrum of Compound **1** | 3 |
| 2 | **Figure S2.** The ^1^H-NMR Spectrum of Compound **1** | 3 |
| 3 | **Figure S3.** The ^13^C-NMR Spectrum of Compound **1** | 4 |
| 4 | **Figure S4.** The HSQC Spectrum of Compound **1** | 4 |
| 5 | **Figure S5.** The HMBC Spectrum of Compound **1** | 5 |
| 6 | **Figure S6.** The HR-ESI-MS Spectrum of Compound **2** | 5 |
| 7 | **Figure S7.** The ^1^H-NMR Spectrum of Compound **2** | 6 |
| 8 | **Figure S8.** The ^13^C-NMR Spectrum of Compound **2** | 6 |
| 9 | **Figure S9.** The HSQC Spectrum of Compound **2** | 7 |
| 10 | **Figure S10.** The HMBC Spectrum of Compound **2** | 7 |
| 11 | **Figure S11.** The HR-ESI-MS Spectrum of Compound **3** | 8 |
| 12 | **Figure S12.** The ^1^H-NMR Spectrum of Compound **3** | 8 |
| 13 | **Figure S13.** The ^13^C-NMR Spectrum of Compound **3** | 9 |
| 14 | **Figure S14.** The HSQC Spectrum of Compound **3** | 9 |
| 15 | **Figure S15.** The HMBC Spectrum of Compound **3** | 10 |
| 16 | **Figure S16.** Cytotoxic activities of compounds 1-4 on PC12 cells at 10 μM | 10 |

**Figure S1.** The HR-ESI-MS Spectrum of Compound **1**


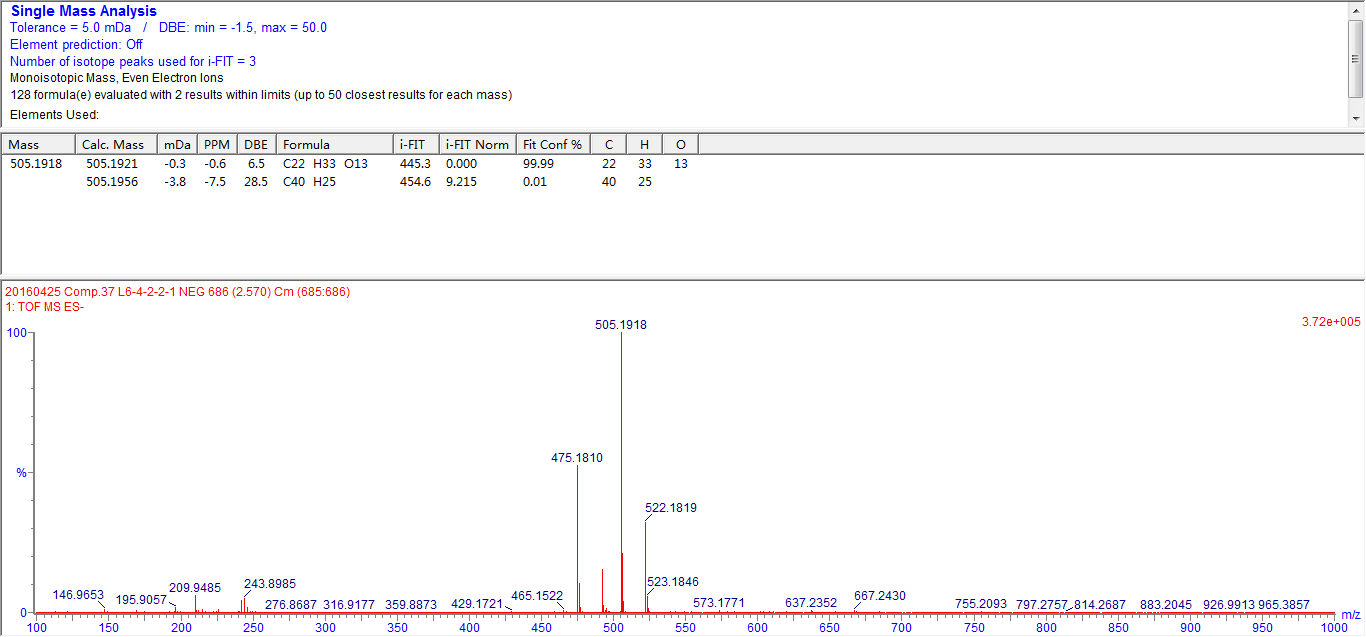


**Figure S2.** The ^1^H-NMR Spectrum of Compound **1**

**
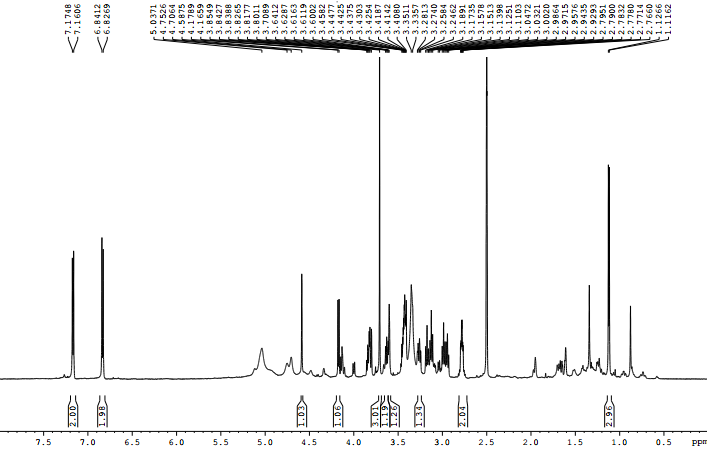
**

**Figure S3.** The ^13^C-NMR Spectrum of Compound **1**

**
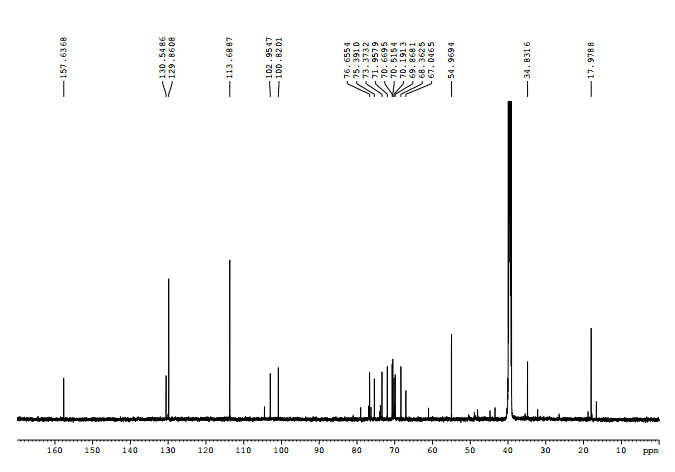
**

**Figure S4.** The HSQC Spectrum of Compound **1**

**
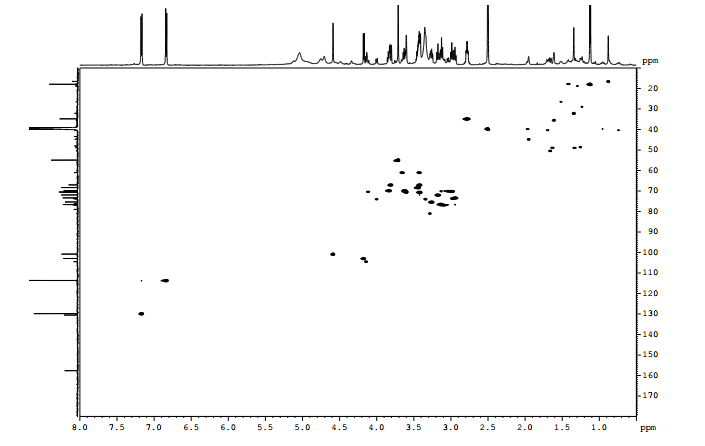
**

**Figure S5.** The HMBC Spectrum of Compound **1**

**
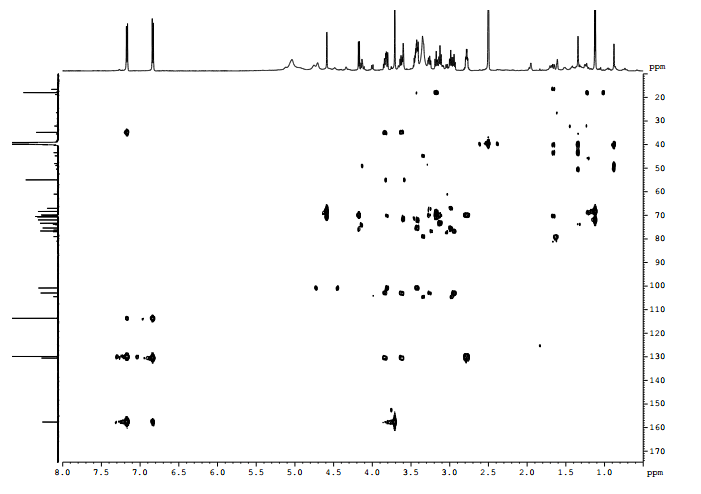
**

**Figure S6.** The HR-ESI-MS Spectrum of Compound **2**


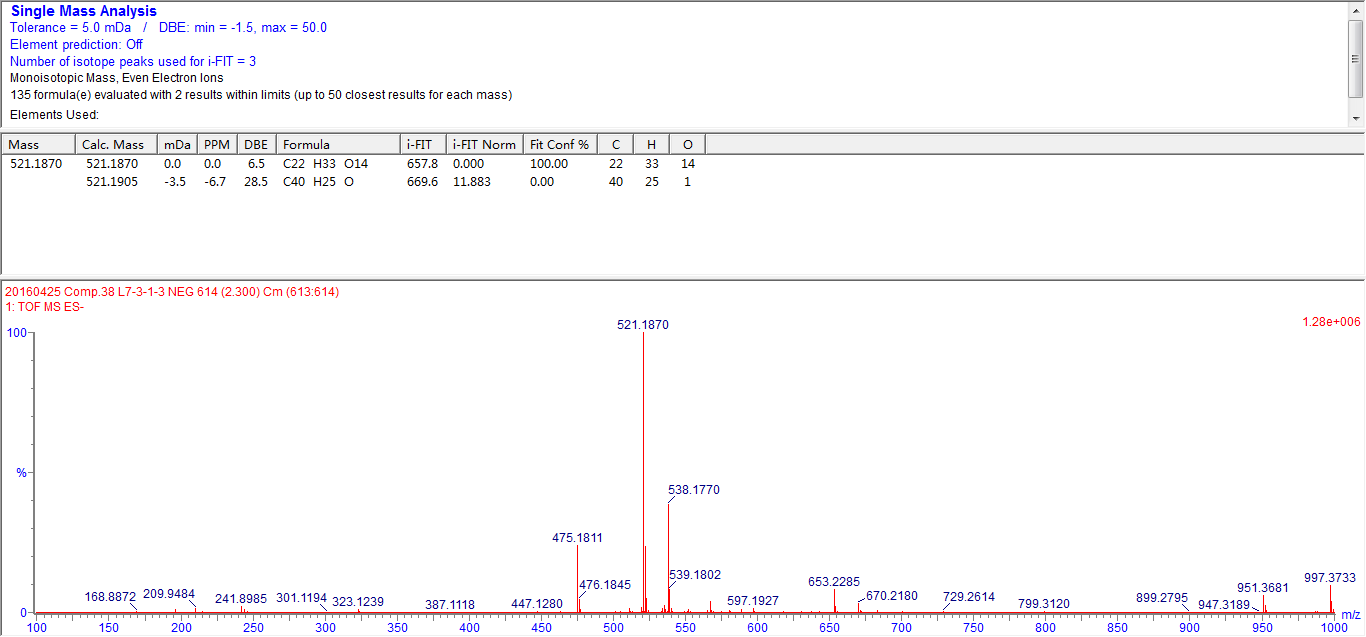


**Figure S7.** The ^1^H-NMR Spectrum of Compound **2**

**
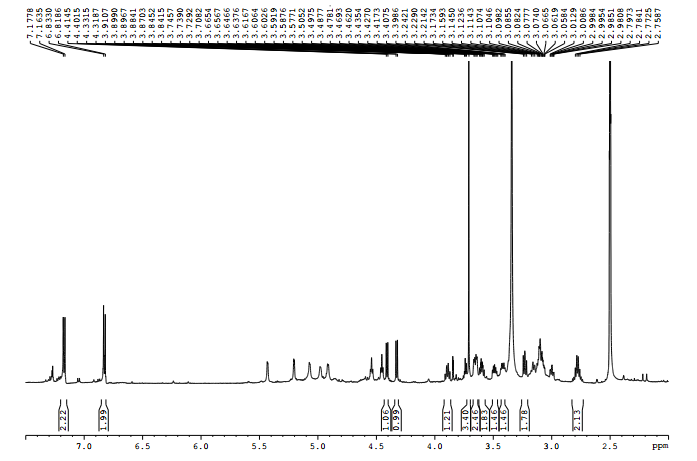
**

**Figure S8.** The ^13^C-NMR Spectrum of Compound **2**

**
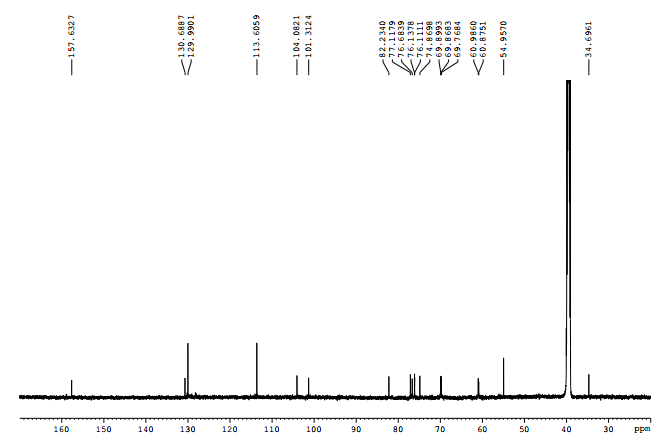
**

**Figure S9.** The HSQC Spectrum of Compound **2**

**
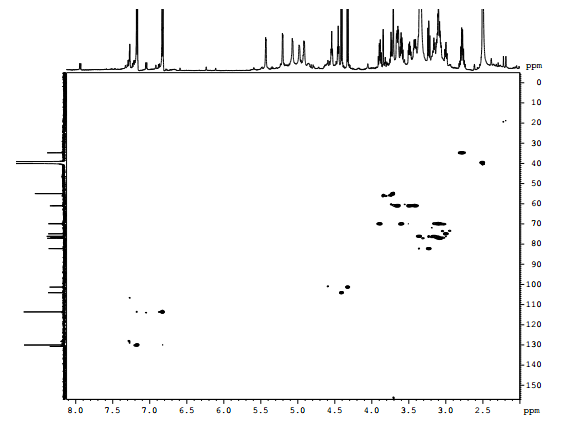
**

**Figure S10.** The HMBC Spectrum of Compound **2**

**
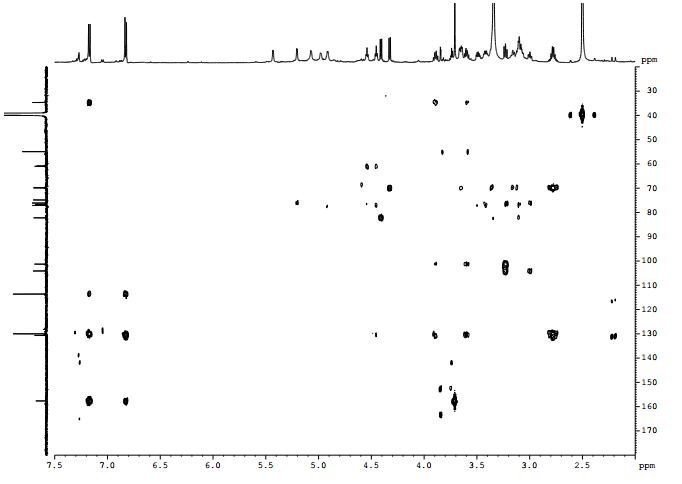
**

**Figure S11.** The HR-ESI-MS Spectrum of Compound **3**


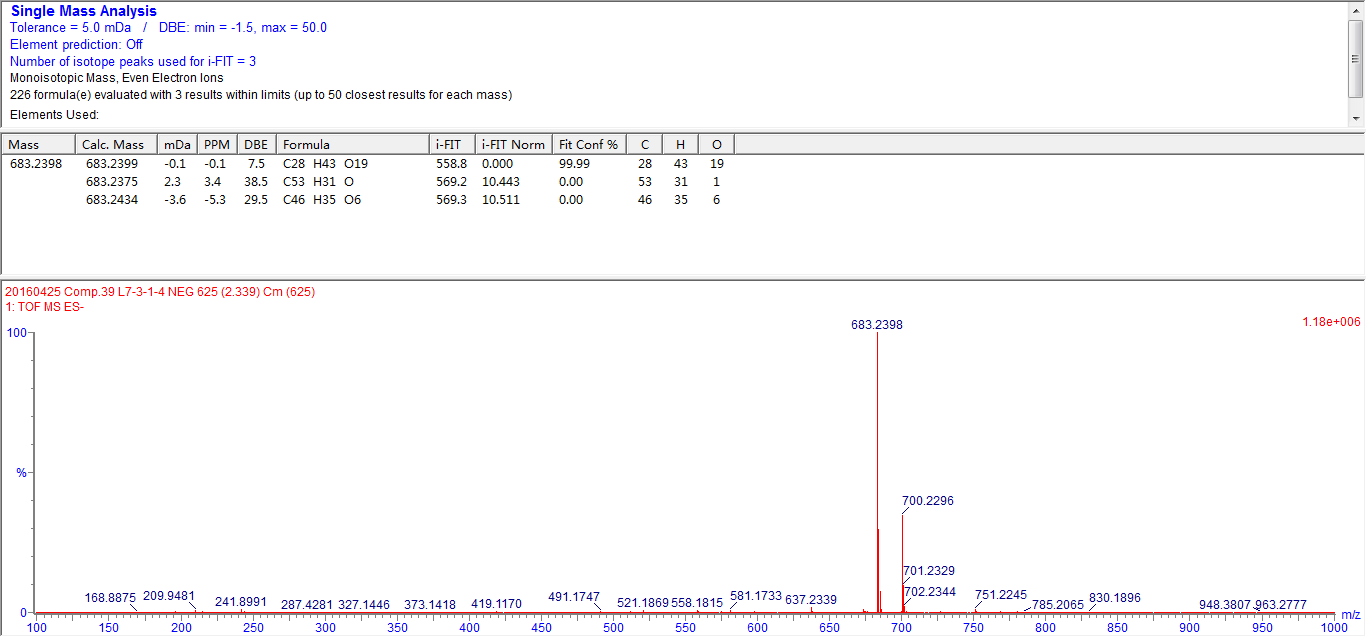


**Figure S12.** The ^1^H-NMR Spectrum of Compound **3**

**
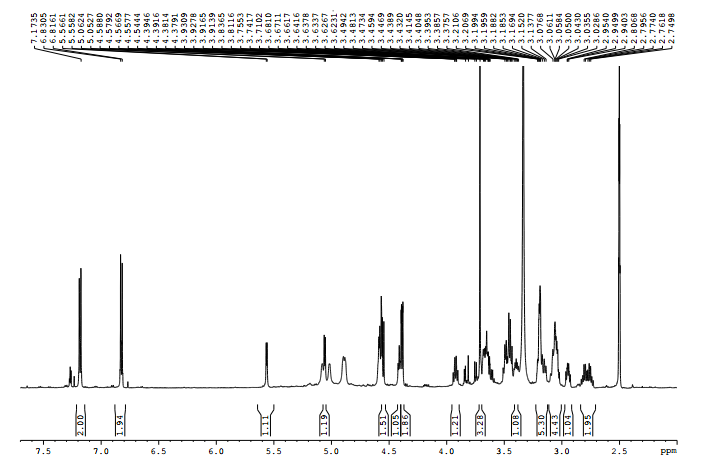
**

**Figure S13.** The ^13^C-NMR Spectrum of Compound **3**

**
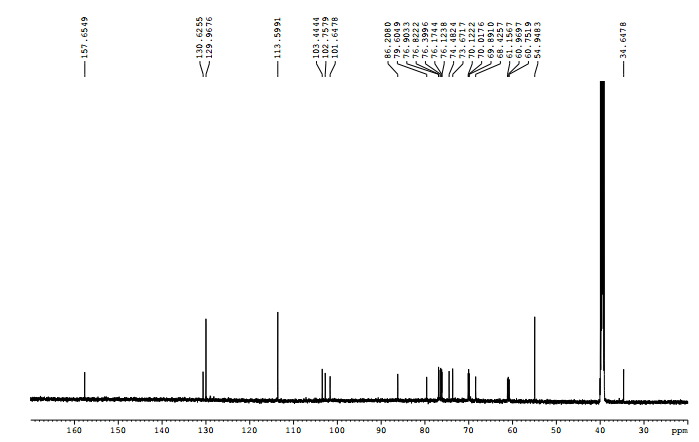
**

**Figure S14.** The HSQC Spectrum of Compound **3**

**
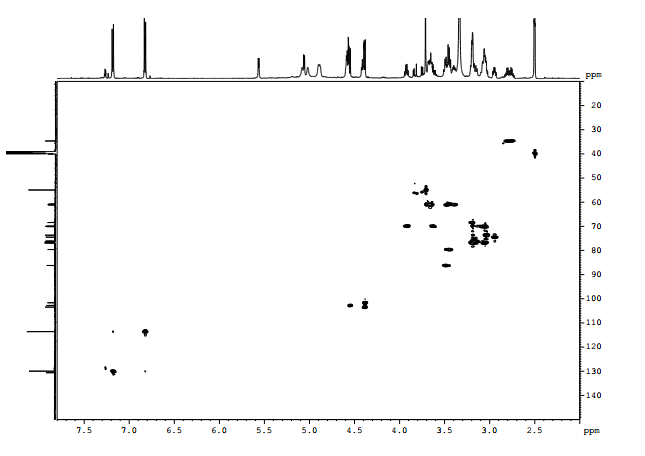
**

**Figure S15.** The HMBC Spectrum of Compound **3**

**
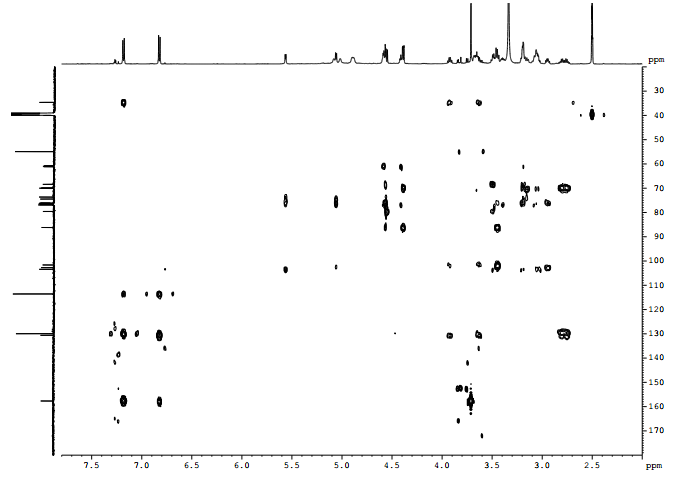
**

**Figure S16.** Cytotoxic activities of compounds **1-4** on PC12 cells at 10 μM
